# Stress process and mental health among agricultural producers

**DOI:** 10.1007/s00127-025-03039-3

**Published:** 2026-01-20

**Authors:** Samantha J. Iwinski, Yifan Hu, Courtney Cuthbertson, Josie M. Rudolphi

**Affiliations:** 1 Department of Human Development and Family Studies, University of Illinois at Urbana- Champaign, 905 S. Goodwin Avenue, Urbana, IL 61801 USA; 2 Department of Agricultural and Biological Engineering, University of Illinois at Urbana- Champaign, Urbana, USA

**Keywords:** Stress process model, Agricultural stress, Mental health, Social support, Resilience

## Abstract

**Purpose:**

Agricultural producers face unique stressors that significantly impact mental health, including depressive symptoms, anxiety, and suicidal ideation. This study offers a novel application of the Stress Process Model (SPM) to examine how agriculture-related stress, social support, and resilience shape mental health outcomes among agricultural producers.

**Methods:**

*N* = 525 Illinois farmers participated in mailed surveys, with data collected in two panels: June-August 2020 (*N* = 296) and March-May 2021 (*N* = 229). Participants completed the Perceived Stress Scale, Farm Stress Survey, Multidimensional Scale of Perceived Social Support, and Brief Resilient Coping Scale. Mental health outcomes were assessed, along with general health. Data was analyzed using structural equation modeling in R.

**Results:**

Higher perceived and agricultural stress were linked to increased depressive symptoms, anxiety symptoms, and suicidal ideation. Social support reduced depressive symptoms (β = –0.121, *p* < .001) and suicidal ideation (β = –0.216, *p* < .001), mediating and moderating the relationship between stress and mental health. Resilience moderated the effects of stress, lowering depressive symptoms (β = –0.100, *p* = .001) and anxiety symptoms (β = –0.088, *p* < .001).

**Conclusion:**

Findings highlight the importance of addressing occupational stress, enhancing social support, and promoting resilience to improve the health of agricultural producers. Interventions should target stress reduction and support systems based on the SPM framework.

 Among the U.S. working-age population, suicide rates have risen by approximately 33% over the past two decades [1] with rates among farmers increasing since 2003 [2, 3]. A recent report ranked the agricultural industry—including sectors like agriculture, forestry, fishing, and hunting—and occupational groups such as farming, fishing, and forestry among the top five for the highest suicide rates [1]. Farmers’ elevated suicide risk may be linked to their unique stressors, lack of personal and social resources, and barriers to help-seeking. Moreover, the deeply ingrained values of self-reliance and dedication to work within farming communities may exacerbate the stress-coping process, making farmers more vulnerable under stressful conditions. Therefore, it is critical to investigate the stress process among farming populations to promote farmers’ well-being.

## Stress process model (SPM)

The stress process model (SPM) is a widely influential framework for understanding how stressors contribute to health outcomes [4–7]. Although the SPM has been sparsely applied in research on agricultural producers [8, 9], its comprehensive approach offers a valuable lens to understand farmers’ unique and persistent stressors. The following literature review is structured to align with the components of the SPM, including stressors, mediators, moderators, and outcomes, as applied to the agricultural context.

The SPM consists of three primary components: sources of stress, mediators or moderators (e.g., personal resources), and outcomes (e.g., mental health). SPM researchers have acknowledged that stress and related processes are shaped by social and cultural factors in which individuals are situated. Farmers operate within a distinct occupational, socioeconomic, and cultural environment that shapes the nature of their stress, their coping mechanisms, and the mental health outcomes they experience.

Stressors in agriculture can be acute (e.g., severe weather, sudden market drops) or chronic (e.g., debt, injuries). Financial pressures, including fluctuating prices, rising debt, and healthcare costs, are major contributors to psychological distress, depression, and suicide risk [10–12]. Occupational demands, such as managing farm tasks and farming’s cyclical nature, add additional stress [13]. Household dynamics and rural isolation in geographically remote areas can exacerbate these challenges [14, 15]. Climate variability and health risks amplify the cumulative burden of these stressors on farmers’ well-being [16–18].

Personal resources—such as coping strategies, social support, and a sense of control—are key mediators in the SPM, shaping how stress affects mental health. These resources, influenced by social and economic contexts, can also change with stress exposure. While mediators link stressors to outcomes, moderators adjust the strength of this link, either buffering or amplifying stress effects. For farmers, a sense of control over operations and confidence in managing crises reduces anxiety and builds resilience [12]. Feeling valued by family, community, and society reinforces purpose, alleviating isolation and enhancing mental well-being [12, 19].

The SPM also highlights the important role of social resources. Social networks, including fellow farmers and industry groups, provide valuable platforms for sharing experiences and coping strategies. Peer support groups and informal networks help farmers connect, while agricultural cooperatives, trade associations, and farmer’s markets offer essential resources, training, and advocacy [12]. Friends within and outside the farming community provide empathy, advice, and a shared understanding of agriculture’s unique challenges [20]. Social support networks are particularly effective in mitigating the negative impacts of stress and improving overall mental health outcomes [21, 22]. Family members contribute significantly by providing emotional stability and security during complex farming operations [22, 23]. However, elevated stress can disrupt family dynamics, complicating these relationships [24, 25]. A recent research synthesis found that social support from partners, relatives, and the community can be both a stressor and a protective factor [26].

Recent literature has highlighted the role of beliefs, values, and meanings in shaping how individuals experience and manage stress [7, 27–29]. For many agricultural producers, farming represents not just an occupation but an ingrained identity tied to legacy and community, making stressors threatening these values particularly impactful. Cultural norms of self-reliance and stoicism, common in farming communities, may weaken personal resources like social support, thereby potentially exacerbating the effects of stress [15, 30–35].

Researchers have also found a strong connection between farming men, masculinity, and help-seeking behaviors. Men who died by suicide were often described as stoic, self-reliant, and action-oriented, yet they tended to avoid medical or mental health support despite facing substantial stressors [26]. A 2020 survey of Iowa farmers revealed that financial health, stewardship motivations, and perceived adequacy of conservation practices significantly impact job satisfaction, while climate change concerns negatively affect it [36]. Agricultural roles shape stress, with women reporting higher stress and lower resilience than men [37, 38]. Women experiencing geographic isolation are four times more likely to report depressive symptoms than their male peers [39].

Accessing support and resources may remain challenging for agricultural producers [40, 41]. Farmers face numerous barriers to accessing mental health care, including cultural norms, stigma, and structural health system issues [30]. Farmers who lived remotely reported worse mental health and well-being than remote non-farm workers, regardless of financial hardship, rural-specific factors, or recent adverse events [17]. Specific barriers that researchers have also found are the need for control of self-reliance, stigma, health system barriers, social image, cultural norms within the farming community, and restrictive lifestyle [14, 15, 17, 30–35, 42, 43]. While not all agriculture takes place in rural communities, the majority does. Residing in rural communities with fewer resources, limited healthcare facilities, and less social integration can exacerbate stress levels among agricultural producers, contributing to feelings of isolation [14, 15].

At the same time, these barriers function as moderators by amplifying the relationship between stressors—such as financial instability or climate variability—and poor mental health. Expanding on the SPM, the beliefs, values, and meanings associated with farming as a way of life further shape how farmers experience and respond to stress. Many agricultural producers view farming as a profession and a legacy tied to family and community roles [44].

The aforementioned barriers not only hinder farmers’ ability to seek help but contribute to the use of maladaptive coping strategies, such as alcohol use, rather than seeking professional mental health support [45]; farmers may have higher rates of alcohol and tobacco use compared to the general population [46, 47]. Farmers often prioritize work over personal health, compounding the issue alongside geographical isolation and limited access to mental health services [14, 15]. While online mental health services show promise, their effectiveness is limited by farmers’ preference for face-to-face interactions and varying technology knowledge [33].

In the SPM, outcomes refer to the mental and physical health effects of stress. Agricultural producers face numerous stressors linked to poor mental health, including financial distress, physical demands, and isolation [48]. The 1980 s U.S. farm crisis underscored the mental health impacts of financial stress, such as depression, lower life satisfaction, alcoholism, and suicide [10, 11, 18]. Reed and Claunch (2020) reviewed U.S. farmers’ mental health, identifying risk factors like pesticide exposure, financial strain, and physical health issues. Occupational injuries [18, 49, 50], high workloads [49, 50], long hours, and government regulations [51, 52] further amplify stress and mental health challenges.

The SPM offers significant potential for understanding how farmers cope with their unique and persistent stressors. While prior research has examined aspects of the SPM concerning agricultural producers’ mental health, these studies have primarily focused on two individual components such as stressors, coping mechanisms, or mental health outcomes. As a result, the full complexity of the stress process in farming populations remains underexplored. This study comprehensively addresses that gap by applying the SPM, integrating its core components—stressors, mediators, and outcomes—to provide a more holistic understanding of how stress impacts farmers’ mental health. Understanding the multifaceted stressors agricultural producers face—including roles, socioeconomic position, family background and structure, and communities—is essential for developing effective support systems and interventions for mental health outcomes.

### Present study

The present study uses the Stress Process Model (SPM) to examine how both agriculture-specific and non-agriculture-related stressors influence mental and physical health outcomes. While previous research has explored stress, coping, and health in agricultural populations, these elements are not always integrated within a formal theoretical framework. Although a small number of studies have referenced the SPM to explain findings in this context, to our knowledge, it has not been directly tested using structural modeling in this population. By formally applying and testing the SPM, this study addresses a critical gap in both the agricultural and stress process literatures. We specifically examine the role of personal and social resources as potential mediators or moderators in these relationships, while controlling for key social characteristics. This approach offers a more theoretically grounded and comprehensive understanding of how stress shapes health among agricultural producers.

## Methods

### Participants

Participants were 525 agricultural producers recruited via mailed surveys from two random samples of 1,000 Illinois farmers. We used simple random sampling from a statewide producer mailing frame to obtain unbiased, generalizable estimates while minimizing coverage and selection bias through a mail-based approach. Of 594 returned surveys, 69 were excluded due to non-involvement in farming or incomplete responses. Sample 1 (*N* = 296) was collected from June to August 2020, and Sample 2 (*N* = 229) from March to May 2021; both samples were combined for analysis. The study was approved by the [MASKED UNIVERSITY] Institutional Review Board, and informed consent was obtained from all participants.

### Measures

#### Stress

Perceived stress was measured by the average score on the Perceived Stress Scale [PSS; [53], which contains ten items on a 5-point Likert scale. Sum scores across ten items represented participants’ perceived stress levels, and the measure showed good reliability (α = 0.87).

#### Agriculture stress

We utilized a modified version of the Farm Stress Survey [54] to assess participants’ levels of worry across seven domains of farming-related stress experienced in the past month. Responses were captured on a 5-point Likert scale. The sum score across the seven items represented participants’ overall agricultural stress levels, demonstrating good internal consistency (α = 0.83).

#### Social support

The Multidimensional Scale of Perceived Social Support [MSPSS; [55] consists of 12 items rated on a 7-point Likert scale to assess perceived social support across three dimensions: significant other, family, and friends. Average scores across 12 items were used for these analyses (α = 0.98).

#### Resilience

The Brief Resilient Coping Scale [BRCS; [56] measured participants’ resilience, meaning their ability to cope with stressful experiences. Participants rated their agreement with four statements on a 5-point Likert scale. The sum score of the four items represented participants’ resilience levels (α = 0.81).

#### Suicidal ideation

The Suicide Behavior Questionnaire-Revised [SBQ-R; [57] includes four items measuring the frequency of suicidal behaviors. The sum recorded score across the four items indicated participants’ level of suicidal ideation (α = 0.80).

#### Depression

We utilized the first eight items of the 9-item Patient Health Questionnaire [PHQ-9; [58] to assess depression symptoms. The final item, concerning suicidal thoughts, was excluded as the SBQ-R already includes suicidal thoughts. Participants rated the frequency of depressive symptoms on a 4-point Likert scale, and the sum score of the eight items was used to indicate participants’ depression symptom severity (α = 0.89).

#### Anxiety

We used the Generalized Anxiety Disorder-7 [GAD-7; [59] to measure anxiety levels. Participants rated their symptoms on a 4-point Likert scale, and the sum score of the seven items was used to assess anxiety symptom severity (α = 0.93).

#### Chronic physical health conditions

Participants were asked to indicate whether they had been diagnosed with any of the following chronic health conditions: asthma, arthritis, cancer (any type), chronic pain (any type), COPD, dementia and/or Alzheimer’s, diabetes, hearing loss, heart conditions, high blood pressure, high cholesterol, or obesity, with responses recorded as “yes” or “no.” A sum score was created of each participant’s total number of chronic physical health conditions.

#### General health

Participants answered a general health question. This question asked participants to rate their overall health on a Likert scale ranging from (5) excellent to (1) poor.

#### Mental health condition diagnosis

Participants were asked two separate questions about whether they had been diagnosed with anxiety or depression, which were included as control variables in the final analysis.

#### Control variables

The final analysis controlled for demographic characteristics, mental health diagnoses, and sample time (sample 1 = 0, sample 2 = 1). Participants reported gender, age, number of children under 18, and 2019 net farm sales (see Table 1). Categorical variables were recoded into binary indicators: gender (male = 0, female = 1), education (above high school = 0, high school or less = 1), marital status (single/divorced/widowed = 0, married = 1), served in active duty (no = 0, yes = 1), and net sales (< $100,000 = 0, > $100,000 = 1). Anxiety and depression diagnoses (no = 0, yes = 1) were also included as control variables.

**Table 1:** Descriptive statistics (*N* = 525)

	*N*	%	M	SD	Range
Age	510		61.31	13.35	22–99
Number of children under eighteen	493		0.52	1.142	0–7
Farming occupation in years	499		36.02	15.904	2–87
*Gender*	503				
Male	452	89.9			
Female	51	10.1			
*Employment*	500				
Full-time agricultural producer	265	53.0			
Part-time agricultural producer, primary income from farm/ranch	62	12.4			
Part-time agricultural producer, primary income from off-farm job	173	34.6			
*Race*	508				
White	508	100			
*Education level*	502				
High school diploma	215	42.8			
Technical or trade school	41	8.2			
Associate degree	89	17.7			
Bachelor’s degree	115	22.9			
Master’s degree or higher	42	8.4			
*Marital status*	514				
Single	33	6.4			
Divorced/separated	26	5.1			
Married	430	83.7			
Widowed/widower	25	4.9			
*Spanish or Latinx ethnicity*	441				
No	440	99.8			
*Current or previous military service*	509				
Yes	80	15.7			
No	429	84.3			
*Net sales in 2019*	434				
≤ $10,000	55	12.7			
$10,000 - $49,999	107	24.7			
$50,000 - $99,999	99	22.8			
≥ $100,000	173	39.9			
*Commodity*	523				
Crop	337	64.4			
Animal	21	4.0			
Both	159	30.4			
Other	6	1.1			

### Data analysis

Little’s MCAR test indicated that the data were not missing completely at random, χ2 (3866) = 4591.95, *p* <.001. Of the 525 participants, 147 had at least one missing response. To handle missingness, we applied multiple imputation with 20 imputed datasets via the Amelia package in R [60, 61]. For each participant with missing values, we then randomly selected one of the 20 completed imputations for analysis to create a final sample of 525 complete cases. We then conducted descriptive analyses and tested the hypothesized moderation and mediation model using the lavaan package [62–64]. To ensure parsimony, only mental health diagnoses (e.g., anxiety, depression) and significant demographic variables were included in the final model.

## Results

### Preliminary analyses

#### Descriptives

The average age of participants was 61.31 years (SD = 13.35). The mean number of years spent in farming was 36.02 (SD = 15.90), ranging from 2 to 87 years. The sample predominantly consisted of males (89.9%), and nearly all participants identified as white (100%). Regarding net sales in 2019, 39.9% had sales exceeding $100,000, and 64.4% identified field crops as their primary commodity. See Table 1 for sample characteristics and Table 2, and 3 for correlation results.

**Table 2:** Correlation coefficients among demographic variables

	Perceived stress	Agricultural stress	Anxiety	Depression	Suicidal ideation	Support	Resilience	General health
Age	−0.22**	−0.22**	−0.24**	−0.14*	−0.11*	−0.01	0.02	−0.04
Education	0.01	0.09*	0.05	0.04	0.14*	−0.05	0.00	0.04
Children in the home (18 and under)	0.13**	0.08	0.19**	0.10*	0.09*	0.06	0.00	−0.02
Years of experience	−0.13**	−0.16**	−0.17**	−0.1*	−0.09	−0.05	−0.03	−0.06
2019 net sales	0.02	0.12*	0.04	−0.04	−0.11*	−0.04	0.04	0.03

** *p* <.01, * *p* <.05

**Table 3:** Descriptives and correlations between model variables

	Variable	M	SD	Sk.	Kt.	1	2	3	4	5	6	7	8
1	Perceived Stress	14.09	6.38	0.52	0.42	1							
2	Agricultural Stress	10.20	5.09	0.15	−0.23	0.65**	1						
3	Anxiety	3.97	4.67	1.50	2.06	0.72**	0.64**	1					
4	Depression	3.58	4.46	1.86	3.55	0.66**	0.59**	0.82**	1				
5	Suicidal Ideation	3.66	1.56	3.47	16.34	0.4**	0.34**	0.38**	0.47**	1			
6	Support	5.44	1.48	−1.22	1.23	−0.21**	−0.09*	−0.13**	−0.19**	−0.20**	1		
7	Resilience	15.04	2.57	−0.29	0.85	−0.38**	−0.17**	−0.33**	−0.2**	−0.14**	0.12**	1	
8	General Health	3.43	0.82	−0.11	0.15	−0.36**	−0.17**	−0.28**	−0.34**	−0.24**	0.06	0.27**	1

** *p* <.01, * *p* <.05

### Main analyses

#### Depressive symptoms

Participants with 2019 net sales over $100,000 reported fewer depressive symptoms (β = −0.069, SE = 0.033, *p* =.036), while those with a depression diagnosis reported more (β = 0.153, SE = 0.041, *p* <.001). Perceived stress (β = 0.322, SE = 0.049, *p* <.001) and agriculture-related stress (β = 0.311, SE = 0.043, *p* <.001) were positively associated with depressive symptoms. In contrast, better general health (β = −0.102, SE = 0.037, *p* =.006) and higher social support (β = −0.121, SE = 0.033, *p* <.001) were linked to fewer symptoms. The model explained 55.4% of the variance in depressive symptoms (R² = 0.554).

Mediation analysis revealed that social support partially mediated the relationship between perceived stress and depressive symptoms (β = 0.032, *S.E.* = 0.012, *p* =.007). Specifically, higher perceived stress was linked to reduced social support (β = −0.265, *S.E.* = 0.065, *p* <.001), which in turn was associated with higher depressive symptoms (β = −0.121, *S.E.* = 0.033, *p* <.001).

**Fig. 1:**
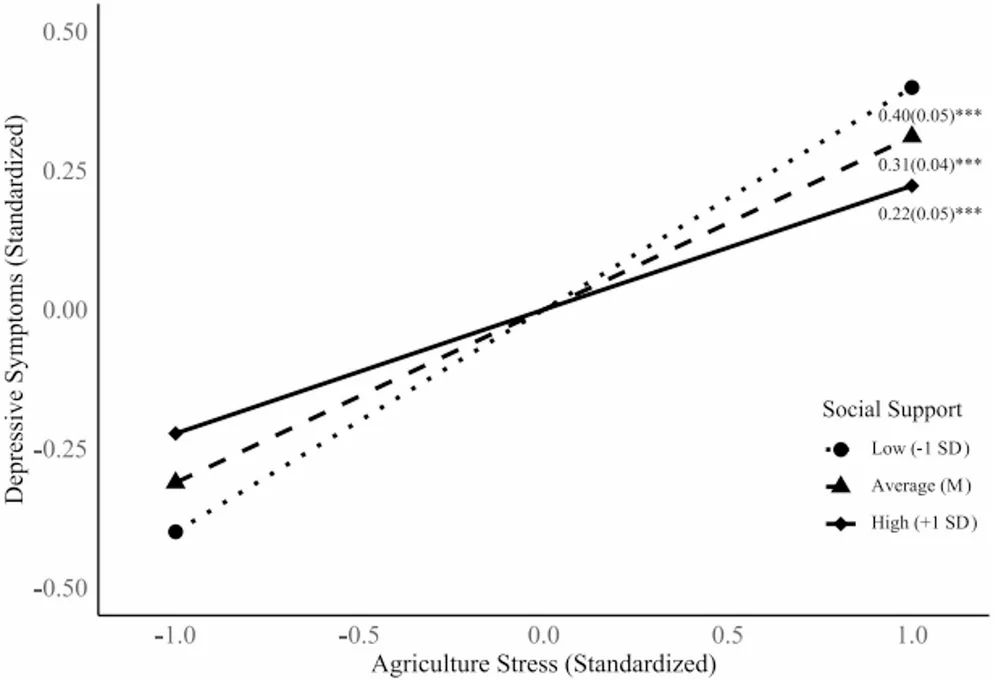
Social Support Moderated the Association Between Agriculture-related Stress and Depressive Symptoms. *** *p* <.001, ** *p* <.01, * *p* <.05

**Fig. 2:**
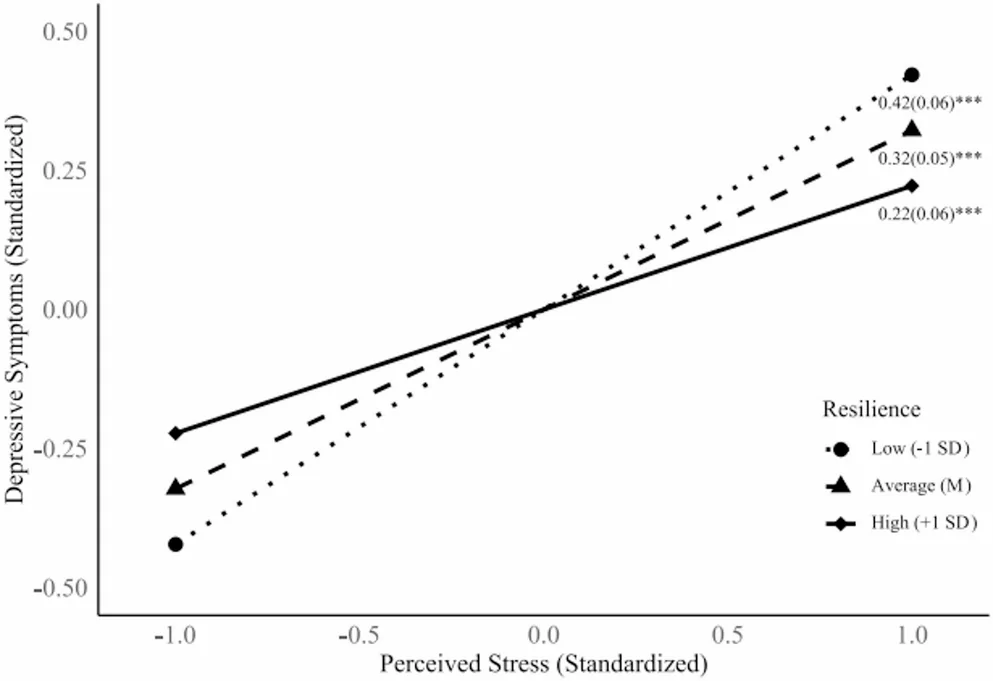
Resilience Moderated the Association Between Perceived Stress and Depressive Symptoms. *** *p* <.001, ** *p* <.01, * *p* <.05

Moderation analyses showed that both social support and resilience buffered the effects of stress on depressive symptoms. Social support moderated the link between agriculture-related stress and depressive symptoms (β = −0.088, SE = 0.029, *p* =.002); the association was weaker at higher support levels. At high social support (+ 1 SD), agriculture stress was still linked to depressive symptoms (β = 0.223, SE = 0.052, *p* <.001), but the association strengthened as support decreased (moderate: β = 0.311, SE = 0.043; low: β = 0.399, SE = 0.052, both *p* <.001; Fig. 1). Similarly, resilience moderated the link between perceived stress and depressive symptoms (β = −0.100, SE = 0.030, *p* =.001). At high resilience, the association was weaker (β = 0.222, SE = 0.057, *p* <.001) compared to moderate (β = 0.322, SE = 0.049) and low resilience (β = 0.422, SE = 0.057), both *p* <.001. This indicates that positive associations were even more positive as resilience levels decreased (moderate resilience level: β = 0.322, *S.E.* = 0.049, *p* <.001; low resilience: β = 0.422, *S.E.* = 0.057, *p* <.001; Fig. 2).

#### Anxiety symptoms

Having more children under 18 was linked to higher anxiety symptoms (β = 0.119, SE = 0.028, *p* <.001), as was having an anxiety diagnosis (β = 0.083, SE = 0.037, *p* =.027). Perceived stress (β = 0.403, SE = 0.042, *p* <.001) and agriculture-related stress (β = 0.319, SE = 0.036, *p* <.001) were both strongly associated with greater anxiety. In contrast, resilience was linked to fewer symptoms (β = −0.090, SE = 0.030, *p* =.003). The model explained 62.5% of the variance in anxiety symptoms (R² = 0.625).

Mediation analysis revealed that resilience partially mediated the relationship between perceived stress and anxiety symptoms (β = 0.037, *S.E.* = 0.013, *p* =.006). Specifically, higher perceived stress was linked to lower resilience (β = −0.410, *S.E.* = 0.057, *p* <.001), and in turn, lower resilience was associated with higher anxiety symptoms (β = −0.090, *S.E.* = 0.03, *p* =.003).

**Fig. 3:**
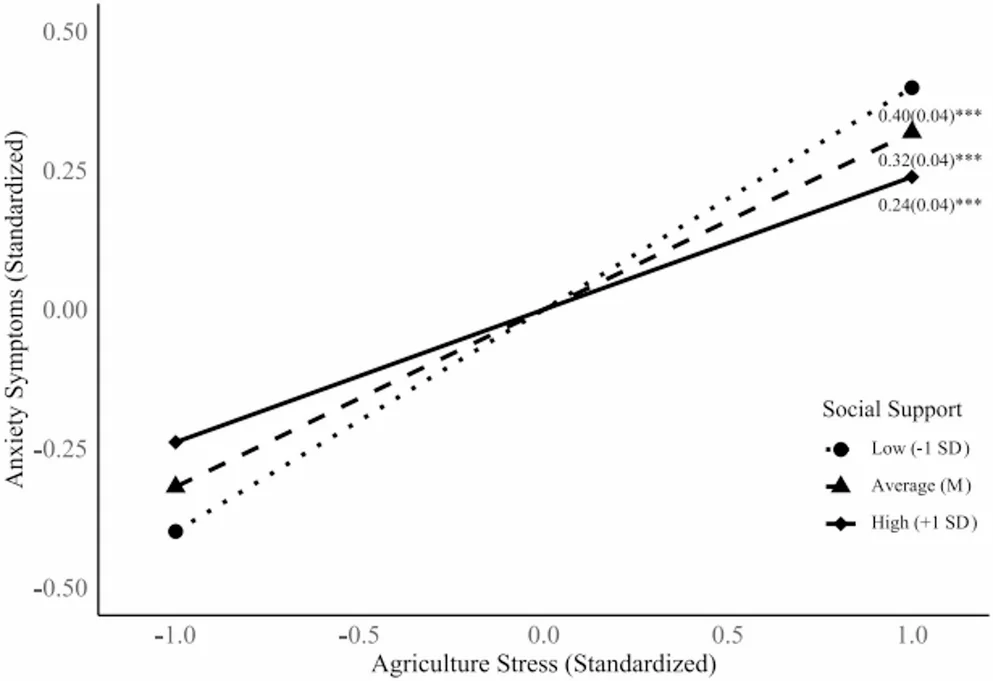
Social Support Moderated the Association Between Agriculture-related Stress and Anxiety Symptoms. *** *p* <.001, ** *p* <.01, * *p* <.05

**Fig. 4:**
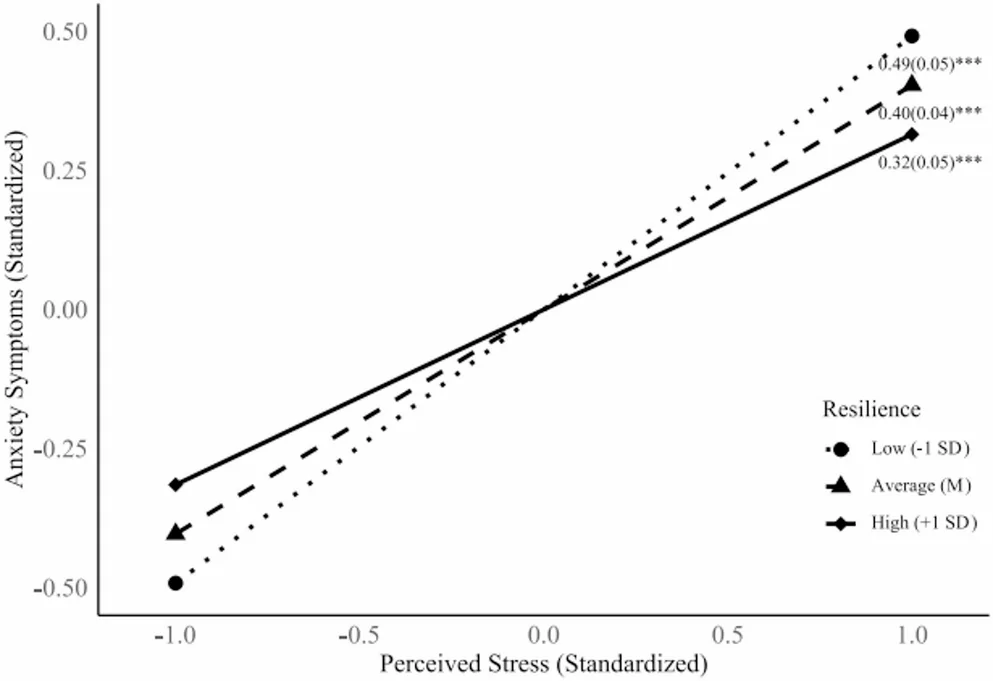
Resilience Moderated the Association Between Perceived Stress and Anxiety Symptoms. *** *p* <.001, ** *p* <.01, * *p* <.05

Moderation analyses revealed buffering effects of social support and resilience on anxiety symptoms. Social support moderated the link between agriculture-related stress and anxiety (β = −0.080, SE = 0.023, *p* =.001), with the association weakening at higher support levels (low: β = 0.399, SE = 0.043; moderate: β = 0.319, SE = 0.036; high: β = 0.239, SE = 0.043; all *p* <.001; Fig. 3). Similarly, resilience moderated the association between perceived stress and anxiety (β = −0.088, SE = 0.024, *p* <.001), with stronger effects at lower resilience levels (low: β = 0.492, SE = 0.048; moderate: β = 0.403, SE = 0.042; high: β = 0.315, SE = 0.048; all *p* <.001; Fig. 4). Resilience also moderated the relationship between general health and anxiety symptoms (β = 0.049, *S.E.* = 0.021, *p* =.023). However, the associations between general health and anxiety symptoms were not significant across low (β = −0.067, *S.E.* = 0.037, *p* =.072), moderate (β = −0.019, *S.E.* = 0.031, *p* =.546), or high resilience levels (β = 0.030, *S.E.* = 0.037, *p* =.419).

#### Suicidal ideation

Females (β = −0.100, SE = 0.047, *p* =.033), those with a high school education or less (β = −0.102, SE = 0.045, *p* =.022), and participants with 2019 net sales over $100,000 (β = −0.090, SE = 0.045, *p* =.044) reported lower suicidal ideation. In contrast, a depression diagnosis was associated with higher suicidal ideation (β = 0.228, SE = 0.055, *p* <.001). Perceived stress was positively linked to suicidal ideation (β = 0.169, SE = 0.067, *p* =.011), while better general health (β = −0.147, SE = 0.049, *p* =.003) and greater social support (β = −0.216, SE = 0.044, *p* <.001) were associated with lower suicidal ideation. The model explained 34.2% of the variance in suicidal ideation (R² = 0.342).

Mediation analysis revealed that social support partially mediated the relationship between perceived stress and suicidal ideation (β = 0.058, *S.E.* = 0.019, *p* =.002). Specifically, higher perceived stress was linked to lower levels of social support (β = −0.268, *S.E.* = 0.068, *p* <.001), and in turn, lower social support was associated with higher suicidal ideation (β = −0.216, *S.E.* = 0.044, *p* <.001).

**Fig. 5:**
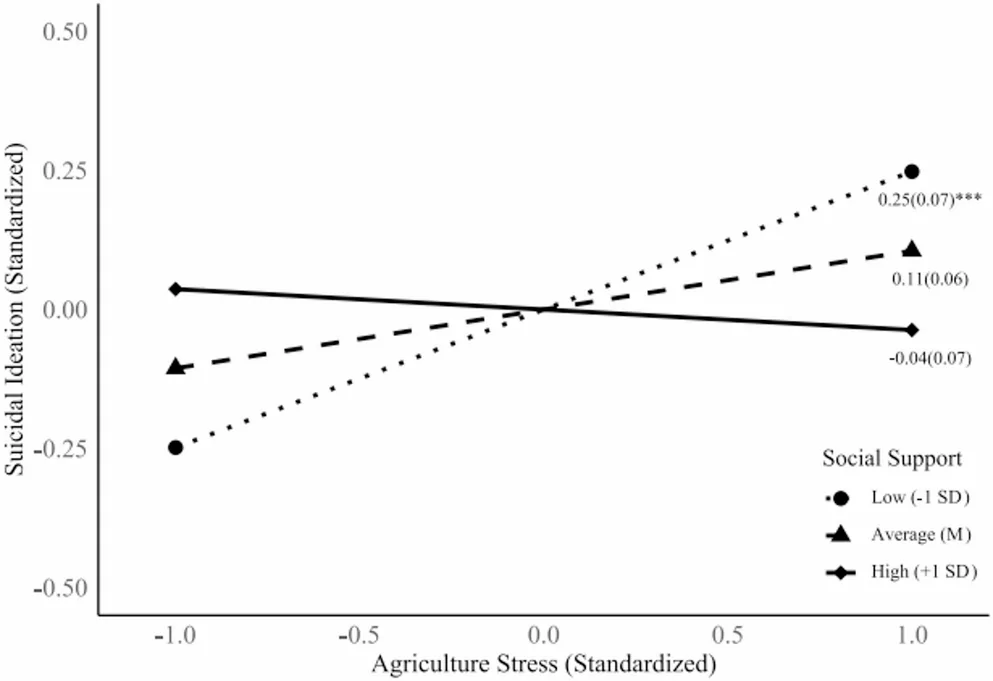
Social Support Moderated the Relationship Between Agriculture Stress and Suicidal Ideation. *** *p* <.001, ** *p* <.01, * *p* <.05

Moderation analyses showed that social support moderated the relationship between agriculture-related stress and suicidal ideation (β = −0.142, SE = 0.038, *p* <.001; Fig. 5). Under low social support, agriculture stress was positively associated with suicidal ideation (β = 0.248, SE = 0.069, *p* <.001), while the association was not significant at moderate (β = 0.106, SE = 0.058, *p* =.068) or high support levels (β = −0.037, SE = 0.069, *p* =.594).

**Fig. 6:**
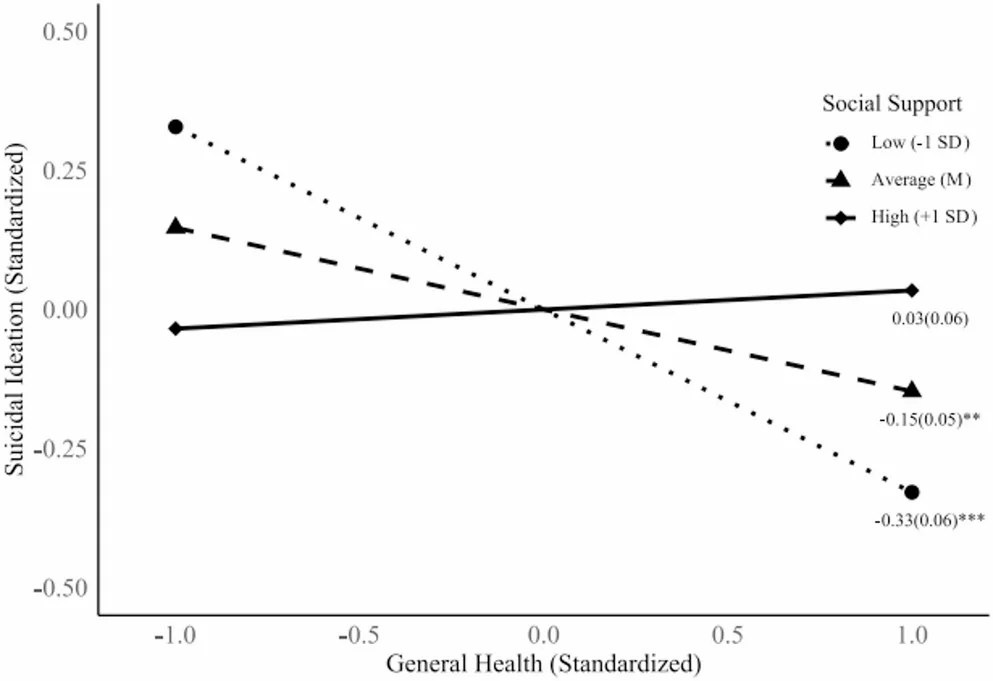
Social Support Moderated the Relationship Between General Health and Suicidal Ideation. *** *p* <.001, ** *p* <.01, * *p* <.05

Social support also moderated the link between general health and suicidal ideation (β = 0.181, SE = 0.038, *p* <.001; Fig. 6). Under low support, better general health was strongly associated with lower suicidal ideation (β = −0.329, SE = 0.062, *p* <.001). This association weakened at moderate support (β = −0.147, SE = 0.049, *p* =.003) and was non-significant at high support (β = 0.034, SE = 0.062, *p* =.580).

## Discussion

This study revealed several significant associations between depressive symptoms, anxiety symptoms, suicidal ideation, and various factors specific to agricultural producers. These findings contribute to the broader literature by reinforcing and extending the application of SPM in agricultural contexts. Our findings affirm this framework by demonstrating the central role of perceived stress and agriculture-related stressors in understanding mental health outcomes. As expected, perceived stress and agriculture-related stress were strongly associated with increased depressive symptoms, anxiety, and suicidal ideation, reinforcing the idea that the unique stressors of agricultural work significantly contribute to mental health challenges [9, 20]. Individuals with depression or anxiety diagnoses experienced more severe symptoms, reinforcing the chronic nature of these disorders [65, 66]. Perceived stress also emerged as a significant predictor of suicidal ideation.

As indicated in the SPM model, personal characteristics and resources influence agricultural producers’ mental health symptoms. Higher net sales were consistently linked to lower depressive symptoms and suicidal ideation, suggesting that greater income may serve as a protective factor for agricultural producers. Demographic characteristics, such as higher anxiety levels among individuals with more children under eighteen, add nuance to the SPM by illustrating how personal and family dynamics contribute to stress exposure. Similarly, lower suicidal ideation reported by females compared to males and by those with less education compared to those with higher education levels invites a deeper examination of the role of social expectations in shaping mental health outcomes.

Resilience, a personal resource, played a critical role in protecting agricultural producers from the adverse effects of stress on mental health. Resilience moderated the relationship between perceived stress and depressive symptoms, indicating that higher levels of resilience enhanced an individual’s ability to manage stress, which in turn reduced the severity of depressive symptoms. This aligns with existing literature and hypotheses, consistently highlighting resilience as a buffer in high-stress environments, especially in professions where stress can be chronic and multifaceted [9, 41]. Resilience was also a significant factor in mitigating anxiety symptoms, with individuals with greater resilience being better able to manage their stress, which in turn reduced the severity of anxiety symptoms. This finding suggests that building resilience could reduce anxiety in agricultural populations, as found in other populations like college students [67]. Some forms of coping and resilience, including positive reframing, life appreciation, existential courage, and engagement in meaningful activities, has been shown to protect against stress, anxiety, and depression during the COVID-19 pandemic, moderating the effects of vulnerability factors on psychological distress [68]. Resilience in farming can encompass various concepts, such as stability [69, 70] and adaptability [71]. However, more research is needed to explore how farmers draw on personal resilience beyond the farm’s resilience.

Aligned with our hypotheses, social support emerged as a vital protective factor across all mental health outcomes in this study, highlighting its significant role in buffering stress and improving mental health. For depressive symptoms, social support was negatively associated with depressive outcomes, reinforcing the findings of previous research that emphasize the value of strong social networks [22, 41]. Notably, social support also mediated the relationship between perceived stress and depressive symptoms, suggesting that people who perceive high levels of stress report lower levels of support, which, in turn, is associated with higher levels of depressive symptoms. This finding reinforces the importance of fostering social ties within agricultural communities, where isolation can manifest as social, emotional, or cultural loneliness [20, 72]. Social support plays a crucial role in reducing the impact of stress on anxiety and suicidal ideation. In the context of anxiety, social support moderated the relationship between agriculture-related stress and anxiety, showing that higher levels of support weakened the positive association between stress and anxiety symptoms, which aligns with the literature [22, 40]. This protective effect was also observed with suicidal ideation, where social support reduced the adverse impacts of perceived stress and agricultural stress. Similarly, social support moderated the association between general health and suicidal ideation, showing that the protective effect of better health was stronger when social support was higher. These findings reflect patterns well-documented in the literature, including the critical role of social support in mental health [21–23, 41, 73, 74], the significant influence of stress on mental health outcomes [12, 48, 75], and the interplay between physical health and mental health in this process [17, 75]. The role of social support is especially significant considering the barriers to help-seeking among agricultural producers, as cultural factors often discourage reaching out for assistance.

The models accounted for 34.2% of the variation in suicidal ideation, 62.5% for anxiety symptoms, and 55.4% for depressive symptoms, indicating that while they explain a substantial portion of the variance in anxiety and depressive symptoms, other factors influencing suicidal ideation remain unaddressed in the current analysis. These findings emphasize the importance of considering additional factors when addressing mental health outcomes among agricultural producers.

This study confirms the value of the SPM in understanding mental health outcomes among agricultural producers while identifying areas for refining the framework. Although updated versions of the SPM acknowledge the role of society and culture, these concepts are not consistently defined or operationalized [76]. Cultural beliefs and community values, which are important in farming populations, remain insufficiently represented in the model. Agricultural producers often internalize cultural norms tied to stoicism, independence, and self-reliance, which can amplify stress and create barriers to seeking help [15, 30–32]. These values are deeply embedded within farming communities and individual identities, often spanning generations. Agricultural stress can represent a dynamic interplay of individual, work-related, and cultural factors shaped by the values and expectations unique to farming communities [34, 35, 43]. Incorporating these dimensions into the SPM would enable a more holistic understanding of how cultural and community dynamics intersect with individual stressors and mental health outcomes. The responsibility of preserving family traditions and land can function as both a stressor and a resource. It may create pressure to uphold a legacy, meet family expectations, and maintain traditional practices, contributing to heightened stress [77]. Conversely, it can foster resilience and motivation, providing a sense of purpose, continuity, and transformation [12, 78, 79]. The generational nature of farming complicates this further, as expectations and values passed down through families shape individual coping mechanisms [80]. Moreover, cultural norms around self-reliance and stoicism may mediate or moderate the relationship between stress and mental health by either intensifying or buffering stress depending on available social support. The dual roles of farmers as both business operators and family caregivers exacerbate these dynamics, with work and home responsibilities often tightly intertwined. Professional stressors, such as financial pressures and climate variability, can be compounded by personal stressors like family obligations and social isolation. This overlap increases the overall burden of stress and complicates the ability to seek support or implement coping mechanisms, as cultural norms may discourage seeking help. These findings underscore the need to adapt theoretical frameworks like the SPM to account for the blurred boundaries between work and personal life in agricultural contexts. Future research should explore the interplay between cultural dimensions and the constructs outlined in the SPM to strengthen its applicability, particularly in rural and farming contexts.

This study highlights the protective role of personal and social resources within the SPM. Interactions between stress, resilience, and social support emphasize the moderating impact of these factors. While stress exposure is a key driver of mental health outcomes, access to supportive networks and effective coping mechanisms significantly mitigate its effects. In alignment with the literature, these findings contribute to rural sociology by demonstrating that social embeddedness remains a critical determinant of mental well-being in rural populations [22, 40]. Notably, the protective role of social support in mitigating suicidal ideation suggests the need to rethink how mental health interventions are designed in agricultural settings. Cultural values of stoicism and self-reliance may limit engagement with traditional mental health services. However, community-driven and peer-based initiatives could offer culturally congruent pathways for intervention, aligning with farmers’ existing social structures [40]. By leveraging social networks and fostering resilience, such interventions could mitigate the mental health burden in agricultural communities while respecting cultural norms.

This study extends the SPM by emphasizing the centrality of perceived stress and occupational stressors in shaping mental health outcomes. Chronic exposure to stressors—particularly in high-risk, unpredictable work environments—compounds mental health risks [5]. These stressors, shaped by both external demands and internal perceptions, interact with personal, professional, and societal factors to create complex, long-term strains that impact mental health. This broader understanding of stress, applicable to various high-stress occupations, highlights the need for a more inclusive framework within the SPM. The SPM has emphasized individual-level stressors, resources, and health outcomes, but our results suggest that within farming populations, these processes are deeply embedded in cultural and community contexts. Cultural values, as discussed, such as stoicism and self-reliance, shape how stress may be perceived, expressed, and managed; however, these dimensions are not well represented in the existing model. The intersection of professional and personal stressors, which can overlap between work, family, and community life, suggests that stress processes in agriculture are collective and individual. To enhance its process, the SPM could be expanded to incorporate culturally grounded constructs, including community norms, shared identities, and intergenerational expectations that can both serve as stressors and as coping mechanisms. Integrating social and cultural mechanisms would extend the SPM beyond its foundations and strengthen relevance within occupational and rural sociological frameworks.

This study has several limitations. Its cross-sectional design limits causal interpretation, and longitudinal research is needed to examine how stress, social support, and resilience influence mental health over time. Although the dataset included two temporal panels, the analyses were conducted cross-sectionally, and measurement invariance across waves was not assessed. As a result, potential differences between waves cannot be fully ruled out, representing a methodological limitation. All variables were assessed through self-reported data, which may introduce recall or social desirability bias. Although anonymity and validated instruments were used to reduce this risk, some level of response bias may remain. The sample, drawn from Illinois agricultural producers, may not represent broader regional, cultural, or economic diversity. The average participant age (61.31) was slightly higher than the USDA-reported average for Illinois farmers (58.6), which may affect generalizability. Additionally, the study did not account for factors such as access to mental health services, cultural attitudes, or economic policy.

## Conclusion

This study’s application of the SPM to agricultural producers advances the theoretical understanding of how occupational, environmental, and psychosocial stressors may interact to influence mental health outcomes in this population. The findings underscore the significance of addressing economic and occupational stressors as critical determinants of mental health, particularly in contexts marked by unique vulnerabilities, such as agricultural work. Social support and resilience emerged as pivotal mediators and moderators within SPM, illustrating their role in buffering the adverse effects of stress on mental health outcomes. The consistent association between higher levels of social support and reduced symptoms across all mental health measures reinforces the theoretical importance of social networks as protective factors. These findings emphasize the need for interventions that foster social cohesion, resilience, and stress management, such as peer support programs and policy-driven efforts to enhance rural social connectivity. These approaches align with the SPM’s focus on the mediating role of personal and social resources in reducing the mental health burden of agricultural producers.

## Data Availability

Due to the sensitive nature of the data, it is not available for public access.
